# Analysing the performance of a health innovation ecosystem in the COVID-19 crisis: complexity and chaos theory perspective

**DOI:** 10.1186/s12961-024-01136-4

**Published:** 2024-05-21

**Authors:** Mehrnaz Moeenian, Sepehr Ghazinoory, Pegah Yaghmaie

**Affiliations:** 1 Department of Technology Management, Faculty of Management and Economics, Science and Research Branch, Islamic Azad University, Tehran, Iran; 2 Department of Information Technology Management, Tarbiat Modares University, Tehran, Iran; 3 School of Business, Capilano University, Vancouver, Canada

**Keywords:** Complexity, Health innovation ecosystem, Edge of chaos, Collaborative networks, Interorganizational systems, COVID-19

## Abstract

**Background:**

This research delves into the complexity management of collaborative networks and interorganizational systems in the health innovation ecosystem on the basis of a best practice in the coronavirus disease 2019 (COVID-19) crisis. The objective is to offer specific solutions and guidelines to stakeholders in the health innovation ecosystem to control the chaos resulting from unexpected events along the ecosystem development and evolution path.

**Methods:**

For this purpose, the performance of the Health Innovation Ecosystem in Iran (the Every Home is a Health Base plan) has been examined through a detailed and in-depth analysis of events and actions taken using documents, reports and interviews with experts. The practical application of chaos and complex adaptive system features (adaptation, time horizons, edge of chaos, sensitivity to initial conditions, state space and strange attractors) is introduced to identify and manage the transition from a state where the health innovation ecosystem is on the edge of chaos and prone to failure. Data were collected through studying documents, reports and interviews with experts, and then analysed using qualitative content analysis techniques, open and axial coding and metaphors derived from complexity and chaos theories.

**Results:**

The findings indicate that to understand and embrace the complexity of the health innovation ecosystem throughout its development and evolution and manage and lead it through the edge of chaos towards successful interorganizational systems performance, it is necessary to use gap analysis to achieve consensus, establish a highly interactive governance structure with key stakeholders of the ecosystem, maintain flexibility to control bifurcations (butterfly effect), prevent transforming emergency solutions into standard routines and ensure the sustainability of the ecosystem against future threats by long-term financial security.

**Conclusions:**

This research provides insights into the dynamics of complex health systems and offers strategies for promoting successful innovation through collaborative networks and interorganizational systems in the development and evolution of the health innovation ecosystem. By embracing complexity and chaos, healthcare professionals, policy-makers and researchers can collaboratively address complex challenges and improve outcomes in health network activities. The conclusion section provides guidelines for successfully managing the complexity of the ecosystem and offers suggestions for further research.

## Background

Health innovation refers to the creation and transfer of new knowledge, methods and approaches in healthcare [1]. Organizations, companies, research centres, universities, hospitals and other institutions active in healthcare fields interact with each other to facilitate healthcare delivery, reduce costs and improve quality of life. This collective group of entities is known as the health innovation ecosystem [2]. In this domain, the activities of organizations are unpredictable and dynamic, while decision-makers must manage complex communications among multiple actors or entities (patients, healthcare providers and suppliers). Researchers emphasize that numerous innovations in the field of healthcare have not only increased their ability to respond to diagnosis and treatment methods, but also have been effective in facilitating more efficient organization [3]. Oldness, population growth and the prevalence of chronic diseases are among the significant factors that increase the demand and costs of the healthcare industry [1]. Therefore, stakeholders in this industry are striving to manage the financial, operational and clinical challenges they will face in the future so that ultimately everyone can benefit from quality, accessible and cost-effective healthcare services. Unforeseen and sometimes unpredictable events, such as the coronavirus disease 2019 (COVID-19) pandemic crisis, challenge healthcare actors in maintaining and developing the ecosystem. Therefore, it is essential to recognize the complexities of relationships and interactions among components, rules and internal and external factors within the innovation health ecosystem and understand its consequences as an adaptive complex system so that we can adequately manage the chaotic conditions that arise from crisis.

Since December 2019, with the onset of the COVID-19 pandemic worldwide and its rapid spread, various countries have been confronted with systemic weaknesses in the health innovation ecosystem (infrastructure, supply chains, policy-making, human resources, public health networks, etc.) that were not adequately prepared to control and combat them [4]. Some low- and middle-income countries (LMICs) such as Iraq, Cuba and Angola were unable to vaccinate even their high-risk populations due to lack of access to adequate health technologies and sufficient logistical infrastructure. In many other LMICs such as Argentina, the Congo and Ethiopia, the focus of the healthcare system on combating COVID-19 has diminished its capacity to provide preventive, screening, treatment and rehabilitation services for non-communicable diseases, leading to a long-term increase in mortality from these diseases [5]. In Bangladesh, Kenya, Nigeria, Uganda and Pakistan, quarantine has led to a reduction in public access, especially in impoverished areas, to healthcare services. This has resulted in a treatment backlog and increased patient waiting lists for urgent health conditions, as well as disruptions in the care and management of chronic diseases [6]. The government of Belarus adopted a policy direction that went against the tide, which involved denying the crisis [7]. Brazil has been facing a gap in its healthcare system due to the lack of adequate and trained human resources [8]. In India, the lack of necessary infrastructure and weaknesses in the supply chain of COVID-related drugs and medical equipment, proportional to the population size, have disrupted the healthcare system [9]. Inadequate and underfunded healthcare infrastructure, coupled with improper organization by government authorities, has been a factor that has caused serious challenges for Romania in dealing with the virus, leading to its widespread and rapid spread [10].

Additionally, the dissemination of misinformation has led to a lack of public trust in health authorities in promoting protective measures such as vaccination campaigns [11]. The unpreparedness of Ukraine’s healthcare system, particularly establishing collaborative networks and necessary ICT-based infrastructure, has resulted in the system’s inability to respond effectively to the pandemic crisis [7]. In Iran, the lack of appropriate vaccines or medicine, the severe shortage of protective equipment and trained human resources and the widespread prevalence of the disease disrupted the health ecosystem [12]. Different countries have implemented strategies and policies to control emergency situations on the basis of their capacities, available resources and geographical, social and economic conditions. Analysing these actions, especially best practices [13], can provide valuable lessons for decision-makers, policy-makers, and actors in the health ecosystem to make better and more timely decisions in the face of future unforeseeable crises.

Since the health innovation ecosystem is a complex, dynamic and nonlinear system [14], the problems in its development and evolution stem from the complexity arising from the interactions and mutual relationships among system entities and their internal and external environment. To address disruptions and crises, it is essential to have a proper understanding and recognition of complexity. The theories of complexity and chaos in the health literature have been of interest to researchers. A variety of theoretical frameworks, empirical studies and practical applications have been conducted to investigate the impact of complexity on the delivery of healthcare services, the role of leadership in managing chaotic environments and strategies for promoting innovation in healthcare systems [15–18]. In defining and understanding complexity and chaos, research has focussed on various theoretical interpretations and models used to explain complexity and chaos, as well as the importance of accepting uncertainty and nonlinearity in healthcare innovation networks. Emphasis has been placed on the need to develop a shared understanding of complexity and chaos to effectively manage these networks [19–21]. The following research area is identifying factors that affect complexity and chaos, in which researchers have focussed on identifying these factors in healthcare innovation ecosystems and examining the roles of various actors, such as researchers, physicians, patients, policy-makers and industry partners. They have also discussed the impact of their interactions on shaping the dynamics of these ecosystems [14, 22].

Moreover, the impact of legal frameworks, financing mechanisms and technological advances on complexity and chaos in healthcare innovation ecosystems has been evaluated. The discussion has focussed on identifying and prioritizing key factors that affect the complexity of healthcare innovation ecosystems. In the field of the impact of complexity and chaos on the outcomes of healthcare innovation, such as the development and implementation of innovative technologies, treatments and healthcare delivery models, research has been conducted to examine the challenges and opportunities posed by complexity and chaos and how they influence the success or failure of healthcare innovation initiatives. The aim is to identify and evaluate the consequences of complex and chaotic environments and develop strategies to enhance innovation [23–25]. The following research area focusses on methods and approaches for managing complexity and chaos in healthcare innovation networks. It emphasizes the importance of enhancing collaboration, structuring robust networks, and improving stakeholder communication to manage the dynamic complexities of these networks. Additionally, the role of leadership, governance and organizational structures in efficient complexity management was examined. The discussion in these studies revolves around the effectiveness of various management models and the need for adaptive and flexible strategies [14, 26, 27]. The last significant research field focusses on emerging trends and future directions in understanding complexity and chaos in healthcare innovation ecosystems and networks and the potential of new technologies such as artificial intelligence and big data analytics to enhance understanding and management of complexity, as well as the importance of interdisciplinary collaborations and integrating different perspectives in advancing knowledge in this field [14, 28, 29]. The focus of discussion in these studies is on identifying research pathways and successful innovations in managing complexity and chaos in healthcare innovation ecosystems and networks.

In this paper, while being in line with previous research in emphasizing the acceptance of complexity by all actors in the healthcare network and examining the outcomes of chaos and complexity and how they affect the network, we analyse the Every Home is a Health Base (EHHB) case in Iran^1^ on the basis of the theoretical lens of the innovation ecosystem and metaphors derived from complexity and chaos theories, and explain the practical application of complex adaptive systems (CAS) and chaos theories in a situation where the health innovation ecosystem faced with crises and disruptions is on the edge of chaos. To this end, we evaluate the problems of the elements and the challenges of managing the complexity of collaborative networks and inter-organizational systems in the innovation ecosystem in this state. Additionally, we highlight the sustainability threats of the health innovation ecosystem on the edge of chaos and re-ordering. The main aim of the article is to provide specific solutions and guidelines so that a health innovation ecosystem can overcome such crises using them and continue its development path at a higher level of order. Moreover, this research differs from previous studies in the following aspects and brings novelty:

- Theoretical perspectives, the research utilizes the ecosystem approach and three characteristics of chaos theory (including sensitivity to initial conditions, state space and strange attractors) as well as three characteristics of CAS﻿^2^ (including time horizons, edge of chaos and adaptation) to analyse the performance of the health innovation ecosystem and propose transition strategies from tipping points to achieve reorganization.
- Without the need to identify the causes of complexity and chaos, it identifies a state in which the healthcare innovation ecosystem experiences disruption and is at the edge of chaos. By analysing short-term changes in the state of the innovation ecosystem, it provides an example for interpreting a sequence of long-term state changes. Thus, it overcomes the two limitations of identifying significant events for determining factors influencing complexity and chaos and requiring historical data.
- The consequences of chaos and complexity were analysed through four outcomes of complex adaptive systems (CAS): bifurcation, emergence, adaptation and path dependency. Our analysis focusses on the path of development and evolution of the healthcare innovation ecosystem, particularly the conditions encountered when it reaches the tipping point (edge of chaos) and the gaps that emerge identified. Then, the construction of a strange attractor for the ecosystem was conceptualized as a solution to navigate through the crisis and fill the gaps. The focus of the discussion is on creating novel collaborative and interactive networks and highlighting the function of interorganizational systems in navigating through the crisis.
- It analyses the role of interorganizational systems in creating collaborative networks. It highlights the function of Information and communication technology (ICT)-based interorganizational systems in the development and evolution of healthcare innovation ecosystems as a solution to overcome the complexity arising from crises.

To this end, in the first section, we present the research background with examples of some developing and underdeveloped countries’ health system challenges faced by the COVID-19 pandemic and categorize previous research on the basis of complexity and chaos theories in the health sector. The second section elaborates on the research methodology. In the third section, research findings are presented in three stages on the basis of the steps outlined in the methodology section. The fourth section discusses the constraints and threats to ecosystem sustainability after transitioning from the edge of chaos. The final section concludes by providing specific guidelines for managing complexity and chaos resulting from crisis conditions for policy-makers and stakeholders in the innovation ecosystem.

## Methods

This research is a case study of the health innovation ecosystem in Iran, the EHHB programme, whose techniques include detailed and in-depth analysis of events and actions taken using documents, reports and interviews with experts. Since we need a deep understanding of the events and related developments in this article, a method of qualitative content analysis has been used to analyse these data [30]. The choice of this method meets the research needs in exploring, describing and explaining how to use CAS and chaos theory to study changes in the health innovation ecosystem. Qualitative content analysis is one of the methods of conceptualization, concept evolution and theory development and is used to address abstraction and conceptual ambiguity.

The process of this research consists of three theoretical stages (literature review), fieldwork (interviews, observations, etc.) and final analysis. In the first step, through the study of literature and research related to complexity and chaos, the theoretical foundations needed for the analysis of this research and guiding semi-structured interviews were obtained. Through documents and reports such as Preparedness and Response for the Control of COVID-19 in the Islamic Republic of Iran, 2020, and GII 2019: Creating healthy lives-the future of medical innovation, the status of the health innovation ecosystem in Iran was examined, and a picture of its situation over four decades (1980–2020) was presented.

**Fig. 1:**
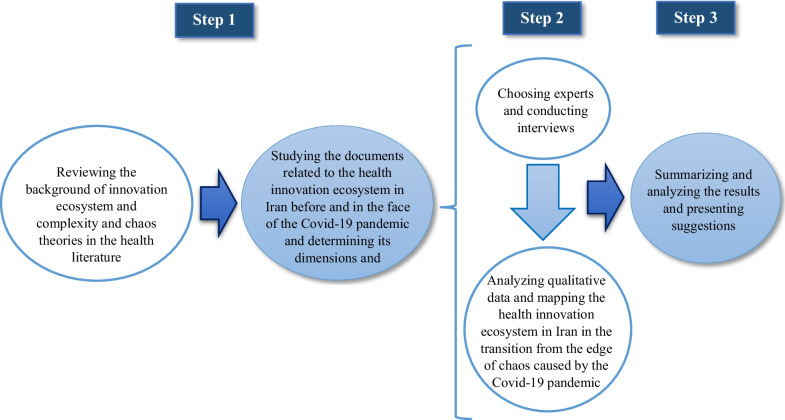
Research implementation steps

In the second step, through one of the qualitative research techniques, namely targeted semi-structured interviews with experts in the health innovation ecosystem and the implementers of the nationwide EHHB programme, the ecosystem’s state during the transition from a  tipping point confronting the disorder resulting from the COVID-19 pandemic was analysed and examined (Fig. 1). Ultimately, in the third step, the above data were analysed using qualitative methods.

### Data collection and processing

In a qualitative perspective, content analysis is a research tool used to determine the existence of specific words and concepts in a text or a series of texts. The researcher analyses the occurrence, repetition and relationships of these words and concepts, then infers the messages within the texts, the authors, readers and even the culture, time and period to which those words and concepts belong [31]. This type of research emphasizes the meanings that involved individuals (participants in the research process) hold about the phenomenon under study [32]. Therefore, to examine the dimensions of the relevant innovation ecosystem more precisely, the opinions of experts in the field were utilized. Purposive sampling method [33] was used to select the samples, and in-depth and semi-structured interviews were conducted with several experts in three categories: health managers and professors of medical sciences; officials of care and support and supervisory teams in the EHHB programme; and NGOs participating in and collaborating with this programme. The duration of the interviews, depending on the circumstances, lasted an average of 45–60 min. The interviews were conducted in Persian and online via video calls, and with the written consent of the interviewees, they were recorded. Data collection continued until saturation, meaning no new dimensions emerged [34]. The number of participants was 15, all of whom held postgraduate degrees or higher, and their work experience was more than 10 years. Then, to complete the information, the primary interview data were combined with secondary data extracted from relevant theoretical studies on chaos and complexity, previous related research in the health innovation ecosystem in Iran from 1980 to 2020, and official reports and news published in the first half of 2020 collected in the first step. Subsequently, breaking down, comparing, allocating, integrating and strengthening the data, theoretical coding was implemented to discover the key concepts related to the function of the EHHB programme on the basis of CAS theory and chaos theory.

To achieve reliability in this research, efforts were made to utilize a conceptual framework as a guide for data collection and analysis. Additionally, interview details, complete implementation of interview texts and the establishment of a database for the research were documented in a way that allowed for examination and tracking of the research process, as well as potential replication by a third party. To assess validity [35], the triangulation approach [36] was employed as a useful tool to enhance the quality of the research. In the triangulation approach, expert opinions were compared with other perspectives, along with documents and observations that were examined. Furthermore, during the research proposal phase, input and feedback from informed individuals or groups were sought to evaluate and provide feedback on the inclusiveness and exclusiveness of the proposed plan. During the data collection phase, after conducting observations and interviews, the text, as recorded and understood by the researcher, was confirmed by the interviewees. Finally, in the data analysis and interpretation phase, two university faculty members and an active manager in this field reviewed the results and findings.

**Fig. 2:**
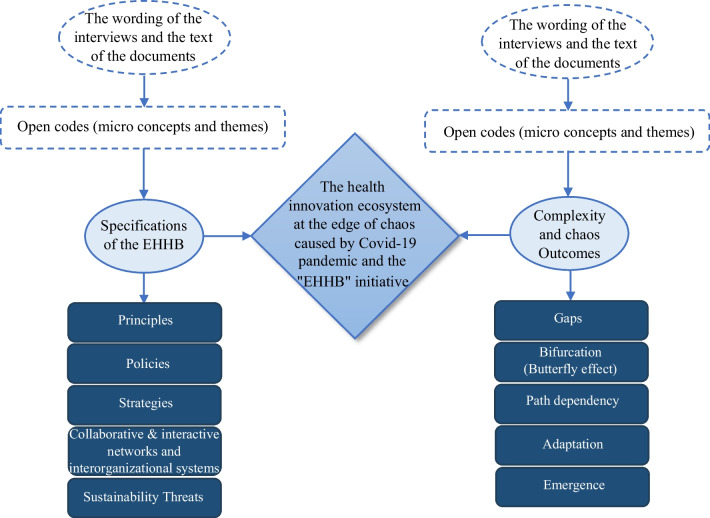
Dimensions of Iran’s health innovation ecosystem in the face of the COVID-19 pandemic

At this stage, all qualitative data obtained from documents and individual interviews were subjected to qualitative content analysis [37]. The qualitative content analysis process included determining the theme, preparing raw data, open coding of the data, coding tables, code categorization, conceptualization, summarization and ultimately establishing a model, map or conceptual framework [38]. The chosen method compensated for the lack of historical data (precise data over successive years) by utilizing storytelling processes [39, 40] and metaphors derived from CAS and chaos theory [41]. Data analysis began by repeatedly reading all textual data to obtain a general sense of the data. Data analysis in the qualitative section took the form of open and axial coding. Some codes, or rather, themes and concepts in this study, were directly derived from the interviewees’ statements, while others were based on concepts and themes drawn from theoretical foundations and literature, empirical evidence, and findings from documents and records, which essentially can be divided into preconceived and emergent codes. Subsequently, on the basis of the conducted examinations regarding the dimensions of the innovation ecosystem, thematic categories were selected, and after data classification and management, verbal propositions in the form of the main dimensions of the Iranian health innovation ecosystem facing the COVID-19 pandemic were determined. These categories, selected on the basis of thematic coding, were chosen from the prominent axes to guide the interviews. Once the thematic categories or codes were identified, open codes were extracted directly from the interviewees’ statements and document texts, essentially related to the interviewees’ statements. After open coding, the next step involved striving to select codes that could represent and cover other codes and give rise to concepts through axial coding. Accordingly, open codes fragmented into specific concepts and themes, while axial codes formed structures or general concepts (Fig. 2).

## Results

On the basis of the explanations provided in the methodology section and in accordance with Fig. 1, the findings of this research are presented in three stages. In the first stage, the results of studying and reviewing research related to complexity and chaos theory and documents related to the health innovation ecosystem in Iran pre- and during the COVID-19 pandemic are presented. These results are used to establish the theoretical foundations required for the analysis of this research and guide semi-structured interviews and explain the dimensions and conditions of the ecosystem. In the second stage, the findings of content analysis of primary data extracted from interviews related to the EHHB case study are presented, and the status of the health innovation ecosystem in Iran in transitioning from the edge of chaos created by COVID-19 is depicted. Finally, in the third stage of presentation, the themes extracted from primary data are combined with secondary data obtained from relevant documents and reports, and key points are identified on the basis of the perspectives of complexity and chaos theories, while outlining the structure of the healthcare network in Iran on the basis of the EHHB plan.

### Stage 1

#### Complexity and chaos theories

Complexity and chaos theories are considered the latest waves of systems theories, which seek to provide fundamental insights into analysing phenomena in the world. Both theories are novel theories rooted in natural sciences but have also expanded into social and human sciences, gaining increasing momentum and utilization by experts in these fields. Complexity theory suggests that understanding and predicting the behaviour of individual components of a system does not necessarily lead to understanding and predicting the behaviour of a complex set of these components [42]. In chaos theory, it is also demonstrated that many known nonlinear systems in physics exhibit unpredictable and chaotic behaviour in such a way that modelling them does not assist in predicting their future behaviour [43]. Complex adaptive systems (CAS) are a particular case of complex systems with many components that simultaneously learn through interactions. CAS creates internal models and uses them to predict the future on the basis of planned actions to achieve expected outcomes [44]. The innovation ecosystem is also considered a complex adaptive system (CAS) that is characterized by constant interactions between different actors [45]. Table 1 illustrates the key features of chaos theory and CAS used in this study to analyse the innovation ecosystem during the COVID-19 pandemic.

**Table 1:** Characteristics of chaos theory and complex adaptive systems (CAS) for case study analysis in this research (self compilation)

Feature	Concept	Application in this paper’s case study
1. Sensitivity to initial conditions	The inability to predict the long-term behaviour of a chaotic system is known as sensitivity to initial conditions. Subtle differences in initial conditions can lead to the success of one innovation ecosystem and the failure of another, even if they are developed identically. Even within an innovation ecosystem, interactions between ecosystem components during different stages of development and different projects can lead to very different outcomes due to subtle variations in initial conditions. Another point to note is that although this sensitivity is defined for initial conditions, this effect can occur at any point in a chaotic system [46]	Given the limitations and difficulties in identifying the causes and outcomes of an innovation ecosystem, by examining observable changes, we can identify patterns and relationships between states and identify factors that may be involved in the emergence or alteration of these states
2. State space	Chaos theory explains the behaviour of chaotic systems by utilizing the concept of a state space, where each dimension represents a variable within the system. The position of an innovation ecosystem in the state space at any given time is defined and determined by the values of the system’s variables. That implies that if the development path of an innovation ecosystem is traced through changes in its state (rather than specific events), it is possible to anticipate when the ecosystem may transition into an unforeseen or undesirable state. With the evolution or change of an ecosystem, its state changes. In chaotic systems, the changes in state represent the trajectory within the state space. These trajectories are random but constrained to a specific region known as a strange attractor [46]	Since observations are limited to innovation ecosystem states, descriptions of status, trends and changes over time are used instead of relying on concrete and historical data. By examining and analysing these descriptions, attempts are made to identify and extract patterns and features of the innovation ecosystem state space in the short term
3. Adaptation	The evolution of an innovation ecosystem as a complex adaptive system requires changes or adaptations in its agents. Hence, the strange attractor captures the evolutionary transformations of the agents as they adapt [47]	The agents of the ecosystem, in their attempt to enhance their adaptability within the environment, exhibit innovative behaviours at multiple levels, ranging from micro-level behaviours (individual agent behaviour) to macro-level behaviours (systemic effects on the entire ecosystem). These behaviours comprise interactions and mutual impacts between the agents and the environment
4. Edge of chaos	The presence of an innovation ecosystem at the edge of chaos (the tipping point) indicates that the ecosystem is in a condition where it has the potential to transition into a new state rapidly. Furthermore, not only is the tipping point the optimal operational point for a CAS, but it also indicates that a CAS can only exist in such tipping points [47]	To better identify and understand the basins and bifurcations within an innovation ecosystem, conceptualizing capacity is crucial. It helps enhance our understanding of the changes in the ecosystem’s state and enables us to identify its optimal functioning at tipping points. Although an ecosystem learns and becomes more adaptable as it evolves, it does not necessarily reach an optimal state of evolution. An ecosystem can get stuck in suboptimal states of adaptation while being attracted to different attractor basins. Therefore, understanding the variables or influential factors and how they change is crucial for comprehending the evolution of the ecosystem and its impact on adaptation
5. Time horizons	The time horizon is of great importance in the evolution of an innovation ecosystem as it determines what kind of decisions and actions its orchestrators should take to promote the development of the ecosystem by considering the adaptation impact duration [48]	Effects of environment and agents will occur in both the short term and long term, and conceptualization should encompass both. Therefore, conceptualizing changes in the state of an innovation ecosystem in the short term provides us with an example for interpreting a sequence of state changes in the long term.
6. Strange attractors	A strange attractor is a path in state space that exhibits the chaotic behaviour of the system over time and its attraction to a small number of ideal states. An ecosystem attractor is an entity, influential agent or state that acts as a stabilizing force and reference point, whereby all ecosystem elements interact and rely upon it to achieve calibrated balance [46]	The strange attractor anticipates the likely future states of an ecosystem and identifies when it will approach the edge of chaos.

The application of each feature mentioned in Table 1 regarding the health innovation ecosystem will be discussed in the subsequent sections of the paper.

#### Health innovation ecosystem of Iran

**Fig. 3:**
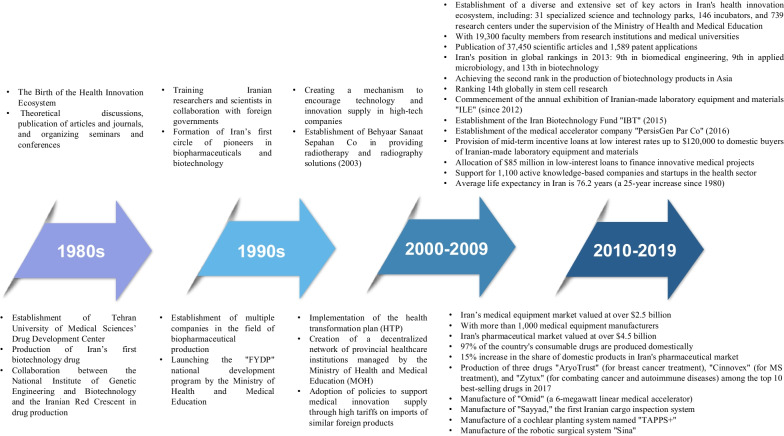
The trajectory of excellence in Iran’s healthcare innovation ecosystem from 1980 to 2020

According to a report by Fitch Solutions, healthcare expenditure in Iran exceeded 35.1 billion dollars in 2018 [49]. On the basis of this, approximately 7.53% of Iran’s gross domestic product is allocated to healthcare expenses [50]. Iran has integrated its healthcare system with medical education to improve its health status, forming 65 medical universities responsible for providing health services and medical education. Together, they form a decentralized network of provincial healthcare institutions managed by the Ministry of Health and Medical Education. Due to its nationwide distribution, this network can conduct endogenous innovation research and train medical personnel on the basis of local needs and epidemic situations [1]. Iran has made significant progress in the healthcare sector in terms of meeting existing needs and demands and the market size over the past four decades. The healthcare innovation ecosystem has been accompanied by the support and backing of regulatory institutions, and in this regard, a set of incentive schemes and policy frameworks have been defined. In addition, the emergence and development of the biopharmaceutical sector in Iran, a highly complex and technologically advanced field, has attracted several successful local export companies. From the perspective of science, technology and innovation [51], considerable efforts have been made to transform the healthcare sector, leading to synergy between human capital development, technological regimes and innovation ecosystems. Figure 3 illustrates the growth and improvement trend of Iran’s healthcare innovation ecosystem from the birth of this ecosystem in 1980 to before the outbreak of the COVID-19 pandemic in 2020 mostly extracted from the report by [52].

The first cases of COVID-19 in Iran were identified on 19 February 2020, when urgent polymerase chain reaction (PCR) tests confirmed four positive cases in the city of Qom. The surveillance system immediately increased its activities for case detection throughout the country. Samples were collected from all suspected cases referred to hospitals and tested for COVID-19 at the national reference laboratory in the Pasteur Institute of Iran. Within 10 days of reporting the first COVID-19 fatality in Iran, cases of COVID-19 were identified in 19 out of the 31 provinces of the country. As of 10 May 2020, the number of laboratory-confirmed cases reported by the national surveillance system reached 107 603, with 6640 fatalities and 86 123 recoveries (Ministry of Health and Medical Education, 2020). By 25 May 2020, Iran ranked 20th among different countries regarding daily confirmed COVID-19 deaths per million people (source: European CDC-Situation Update Worldwide).

Iran is a developing country with a large population of more than 80 million people and a diverse cultural and ethnic makeup. It is also subject to international sanctions, which affect its international relations. In addition, the COVID-19 pandemic had unique and unprecedented circumstances due to being widespread as well as its uncertainty and speed, which set it apart from other crises such as floods, earthquakes or even contagious diseases such as cholera. There was no predetermined tool or formula for controlling such a crisis, and responsible institutions alone could not succeed in managing the situation; agile and practical strategies needed to be adopted. Public awareness of the dangers of a crisis at the right time can be an excellent way to save the lives of those in trouble. Therefore, social participation throughout the country was essential [53].

**Fig. 4:**
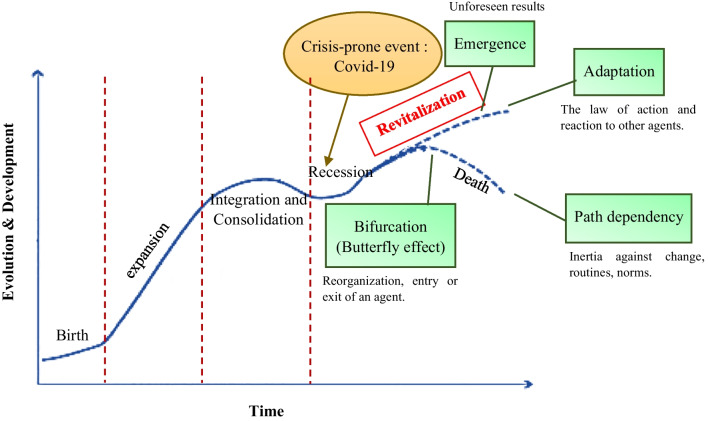
Development stages of Iran’s healthcare innovation ecosystem until the first half of 2020 (self compilation)

According to the information provided regarding the development and evolution of Iran’s health innovation ecosystem from 1980 to 2020 (Fig. 2), this ecosystem has undergone stages of growth and development from the birth stage to integration and consolidation over four decades. It has been shaped by extensive collaboration networks across the country, consisting of various actors, including governmental and legislative institutions and private and public healthcare sectors such as hospitals, clinics, medical complexes, laboratories, pharmaceutical companies, pharmacies, universities, research and development centres, accelerators, startups and knowledge-based companies, among others. In the first half of 2020, with the emergence of the COVID-19 pandemic, the development trajectory of this ecosystem was disrupted, and the conditions of the ecosystem underwent a significant change, leading to an imbalance and a shift towards a recessionary state. This state change has bifurcated the development trajectory of the ecosystem, creating a butterfly effect, and transitioning from the edge of chaos to either a state of revitalization or decline is contingent upon the correct diagnosis and response by ecosystem actors to reorganize, establish interorganizational systems, and create new collaborative and interactive networks (Fig. 4).

### Stage 2

#### Analysing Iran’s health innovation ecosystem at the edge of the COVID-19 chaos

The edge of chaos is a narrow transition zone between order and chaos that facilitates the emergence of new behavioural patterns, such as innovation and self-organization [54]. Being at the edge of chaos is an essential feature of CAS for innovative ecosystems, as it indicates that the ecosystem is in conditions where it can rapidly transition to a new state. Additionally, not only is the edge of chaos the optimal operational point for a CAS, but it also signifies that a CAS exists only at such tipping points. Changes in a CAS also lead to the emergence of co-evolution within its environment. Co-evolution at the edge of chaos means that agents evolve together and simultaneously adapt to increase their fitness, creating a dynamically changing adaptation landscape.

If the state of the ecosystem and the strange attractor are unknown and unfamiliar, the change may manifest as an unexpected outcome. It is also possible that there are factors that allow the ecosystem to react quickly to environmental changes and optimize its performance. The presence of an ecosystem at the edge of chaos is also defined as the capacity for action of the system, indicating that ecosystems without this capacity suffer from inertia, leading to their failure [55]. In other words, when systems operate at a tipping point (the edge of chaos) and undergo evolution, a small and relatively insignificant change can cause the system to transition into a completely different state rapidly [56]. This can signify the difference between success and failure. Different evolutionary states are regions in the strange attractor known as basins of attraction. A basin of attraction is a configuration where a system tends to persist. Strange attractor properties used by altering the environment to maintain the system in an optimal state or to bifurcate it into a new state. Therefore, understanding the variables or influential factors and how they change is utilized for comprehending the mechanisms of ecosystem evolution. Strange attractors, tipping points (edge of chaos) and bifurcations are essential features of chaotic systems for innovation ecosystems, as the evolving chaotic innovation ecosystem may quickly diverge into different states. In addition, planners and managers may be confused about what is happening. This is the very state that Iran’s health innovation ecosystem was experiencing in early 2020. The sudden state change of ecosystem due to the COVID-19 crisis created imbalances and led to the emergence of gaps between the ecosystem’s components. The phase transition at this tipping point can lead to either failure or success, depending entirely on choosing the right strategy in dealing with the appropriate strange attractor that can guide the ecosystem towards the basin of attraction that ensures its survival. In these circumstances, the ecosystem orchestrators recognized that using social participation in the form of the social innovation project EHHB was the strange attractor that the ecosystem needed on the edge of chaos.

**Fig. 5:**
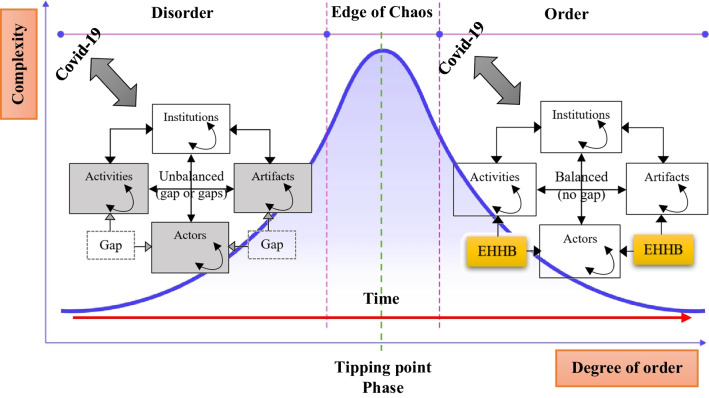
The state change of the innovation ecosystem in Iran’s healthcare sector at the edge of chaos in the first half of 2020 (on the basis of the state space process model derived from chaos theory) (author compilation)

The nationwide EHHB programme was designed by the Ministry of Health and Medical Education with a neighbourhood- and family-centred approach to inform and control the pandemic. The main objective of introducing this idea in the first half of 2020 was to expand the network of collaboration and increase interaction among stakeholders in the country’s healthcare ecosystem without vaccines, medications and sufficient resources to control the pandemic. In this regard, they employed all national capacities, especially NGOs, to assist the Ministry of Health and Medical Education in controlling COVID-19 by entrusting control to local communities by establishing coordination and managing active units in neighbourhoods (health bases in urban areas/health houses in rural areas) and engaging other stakeholders, such as the Red Crescent, as an appropriate strategy for disease control at the community level. The approach to engaging the public in this plan involved selecting a health ambassador for each household and a neighbourhood health intermediary for every 40 households (ambassadors chosen from NGOs), with a focus on mobilizing community resources and improving the social, economic and cultural conditions of the community. Figure 5 illustrates the state change of Iran’s health innovation ecosystem on the edge of chaos by the aid of EHHB programme in the first half of 2020 on the basis of a state space process model derived from chaos theory.

For example, in the city of Kouhdasht, a district of Lorestan province with a population of more than 89 091 individuals, 89 care teams, 90 monitoring teams and 23 support teams were deployed, involving a total of 434 personnel. Due to some patients lacking the ability and conditions to visit medical centres, doctors and nurses provided healthcare services at the patients’ homes. Additionally, by forming 14 care teams from NGOs, they disinfected people’s homes. On this basis, the county centre was divided into seven blocks, each assigned to health centres and NGOs located in that block. Prior to implementing the EHHB plan, approximately 30–40 residents of Kouhdasht were hospitalized daily, but after implementing this plan, the number decreased to an estimated 3–8 individuals per day, alongside a reduction in COVID-19-related mortality in the region.

### Stage 3

#### The dimensions and structure of Iran’s health and treatment network based on the EHHB plan

The EHHB plan is a prominent example of inter-organizational system coordination, bottom-up planning, optimal use of available resources and the creation of collaborative networks in the healthcare ecosystem. The dimensions of the EHHB plan, extracted from open codes (micro concepts and themes), are summarized in the form of principles, policies and strategies in Table 2.

**Table 2:** Dimensions of the EHHB programme extracted from open codes

Principles	Policies	Strategies
• Involvement of NGOs • Neighbourhood- and family-centric approach • Alignment with the healthcare network system • Mobilizing public participation • Adherence to infection control measures • Provision of active services and care • Promoting interdisciplinary collaboration • Utilizing innovative technologies	• Providing essential healthcare services (in-person/remote) • Early identification of COVID-19 and individuals at high risk of exposure (households/workplaces) • Home/non-home quarantine and care for at-risk groups • Development of outpatient treatment • Improving the quality of inpatient care	1. Strict monitoring of health protocols and law enforcement 2. Widespread, targeted and intelligent PCR testing 3. Active case finding, quarantine and intelligent tracking 4. Education, information dissemination and cultural promotion 5. Protection of vulnerable groups, including older adults and those with underlying conditions 6. Smart and targeted determination and announcement of restrictions 7. Strengthening outpatient treatment processes and minimizing hospitalizations

The important task of planning and coordination for public education to enhance people’s knowledge and skills in dealing with COVID-19 is entrusted to the Central Committee, under the responsibility of the Provincial Medical University. The EHHB programme relies on local resources and the extensive participation of community organizations and the public. It has entered the implementation phase through support, care and supervisory teams. These teams have created special collaborative and interactive networks that utilize information and communication technology tools, including telephone systems, internet portals, verbal screening and the Mask app to develop interorganizational systems. These networks helped facilitate the transition of the health innovation ecosystem from the tipping point and existing gaps in various layers and acted as a strange attractor.

- *Support teams:* with a focus on NGOs, public participation and the collaboration of household health ambassadors and community health intermediaries, they were responsible for distributing public and organizational assistance to vulnerable and high-risk populations.
- *Care teams:* with the participation of NGOs, two-person teams consisting of household health ambassadors and community health intermediaries responsible for identifying infected individuals and caring for people in close contact with infected individuals were formed.
- *Supervisory teams:* with the focus on environmental or professional healthcare experts as the team leaders and volunteers from the Red Crescent and NGOs (groups of 4–5 people), they were responsible for monitoring service centres, procuring and distributing food supplies and overseeing the existing industrial and trade units in the neighbourhood.
- *Telephone systems:* The telephone hotline system was responsible for guiding and educating people about prevention and personal care, necessary care for pregnant mothers, mental health, healthy nutrition during the COVID-19 pandemic and providing information about specialized centres.
- *Internet portals:* the internet portal allowed individuals to self-assess COVID-19 symptoms and receive guidance on further actions if they experienced positive symptoms. If they had an electronic health record or were under the care of a relevant healthcare provider, they were contacted. After reassessment, necessary instructions were given, and the process of care and treatment was explained in detail.
- *Verbal screening and follow-up:* this was conducted by every healthcare provider or health monitor as soon as they were informed of a positive COVID-19 test result for any individual in their covered population (either 700 rural residents or 2500 urban residents) through the electronic health record system. They contacted the affected individual to ensure compliance with isolation or home quarantine measures and provide health recommendations according to the communicated guidelines. They would also inform the contact tracing coordination team to prevent the possible spread of the disease by tracking close contacts of suspected and probable individuals across the city.
- *The Mask app:* by installing this app on their smartphones, patients receive their COVID-19 test results through the Mask app. In addition, individuals could receive instructions on prevention, care, personal protection, living with coronavirus and caring for a patient with COVID-19 through the app.

The first step in implementing the programme was to establish regional divisions and organize human resources according to the predetermined execution teams. This was planned on the basis of the levels of primary healthcare provisions in rural and urban areas. All cities were divided into regions on the basis of defined population criteria, and one person was designated as the responsible individual for each part. Ideally, they were stationed at selected comprehensive health service centres for COVID-19, taking on the responsibility of managing the programme. In small cities, each region is considered to have an average population of 40 000 people, while in larger cities, it was estimated to be 80 000 people per region. In metropolitan areas, the figure is set at 120 000 people per region. In the first half of 2020, approximately 11 134 290 health ambassadors at the household level and 280 619 neighbourhood health connectors were collaborating in implementing the EHHB plan.

**Fig. 6:**
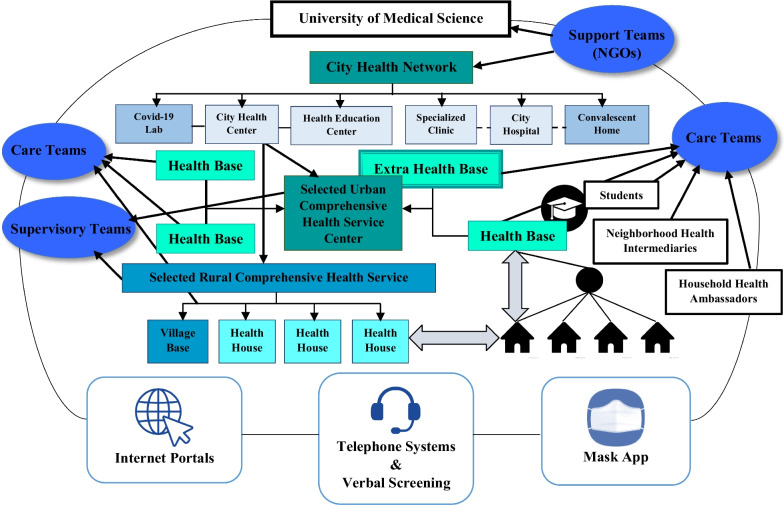
Iran’s healthcare network structure during the COVID-19 pandemic based on the EHHB initiative

In the designated area, organization and planning were carried out for the activities of operational committees and execution teams with joint management and participation of NGOs and selected comprehensive health service centres. In rural regions, organization and planning are carried out with a focus on rural comprehensive health service centres and health houses, with the participation of NGOs. Furthermore, efforts were made to involve residents and volunteers from the same village in providing services to rural communities (Fig. 6).

Statistics have shown that in areas where the programme was fully implemented, the screening speed was twice as high as that in other areas. Health ambassadors played a significant role in adhering to COVID-19 health protocols, producing masks, assisting those in need and emphasizing intelligent physical distancing in communities, leading to noticeable impacts (Table 3).

**Table 3:** Achievements of the social innovation project EHHB for the health innovation ecosystem in Iran (based on: Ministry of Health and Medical Education, 2020; additionally, Iranian official newspapers and websites)

• The chain of virus transmission cut off by increasing adherence to health protocols and active tracking and monitoring by at least 80% in 1 month and 90% in 3 months
• Hospitalization rates reduced by at least 30% in 1 month and 50% in 3 months
• Mortality rates reduced by at least 10% in 1 month and 40% in 3 months
• Supportive coverage provided for at-risk groups by 50% in 1 month and 90% in 3 months

Three key points of the EHHB plan analysis have been identified on the basis of the perspective of complexity and chaos theory that the Iranian healthcare ecosystem has utilized to embrace complexity and transition from a chaotic situation and has contributed to the success of the plan.

i. Acknowledging complexity through identifying and analysing gaps
  Observing the noticeable changes in the state of the health innovation ecosystem (Table 1/Feature 1), the Iranian health network has realized that due to the unknown nature of the virus and its spread rate and mechanism, the current processes and systems are not aligned with the increasing volume of COVID-19 cases and related mortality rates. It has led to an increase in the density of requests for COVID-19 testing (PCR) and hospital admissions, far beyond the capacity for acceptance and care, adding complexity to pandemic planning and control. By describing the states, trends and changes in the short-term time frame (first half of 2020), the agents analysed at the micro-level and by identifying patterns and relationships between states, gaps that may be involved in the emergence or change of these states have been identified (Table 2/Feature 2). The identified gaps in different layers of the ecosystem were as follows:
  *Artefact:* The lack of suitable vaccines or medications, severe shortages of disinfectants, masks and diagnostic tests (PCR). Innovation is needed to quickly address these shortages and alleviate the pressure they cause.
  *Activities:* The roles and behaviours within Iran’s healthcare innovation ecosystem at the discussed tipping point could not guide the ecosystem towards crisis transition. Therefore, defining new roles and corresponding activities was essential. In other words, various types of complex nonlinear relationships need to rapidly emerge in different stages of interactive activities among ecosystem actors.
  *Actors:* Given the development trajectory of the health innovation ecosystem in Iran (Fig. 3), actors with diverse natures, internal processes and levels of evolution/maturity were present in this ecosystem. However, the acute crisis had such extensive dimensions that without the addition of new actors, including non-specialists (NGOs and public participation), transitioning from the situation would have been extremely difficult, if not impossible.
  The outcomes of these gaps were the need for new actors in the health network (Fig. 4: bifurcation) and routine changes based on the conditions created by the pandemic (Fig. 4: path dependency). Therefore, if timely and effective communication had been utilized to engage public participation in pandemic control, it could have assisted the existing system in recovering from the butterfly effect (bifurcation) and guiding the ecosystem from recession (Fig. 4) at the tipping point (Table 1/Feature 4) towards resilience and equilibrium. Recognizing the optimal functioning of the innovation ecosystem at the edge of chaos requires a correct understanding of its capacities during times of state change.
ii. Embracing complexity and establishing a highly interactive management system
  During the gap analysis, a governance structure was developed for the programme. This structure kept the programme on track and ultimately made its success possible. For precise and coordinated implementation of the plan, coherence in planning and execution coordination was crucial. All decision-making and actions were taken within the framework of the national categorization (minister of health and medical education, minister of communications and information technology, etc.), provincial level (governor-general, presidents of the medical universities, director-general of the Red Crescent, etc.), county level (governor, head of the health and treatment network in the county, mayor, etc.), regional level (mayor of the region, head of the regional health centre, Red Crescent representative, etc.), district level (district mayor, head of comprehensive health services centre, Red Crescent representative) and neighbourhood level (technical supervisor of health base, health house manager, representative of NGOs organizations). These decisions and actions were implemented following the national healthcare network system.
iii. Adaptability to complexity and emphasis on flexibility to maintain the dynamism of the ecosystem
  To ensure the successful implementation of the EHHB initiative and manage the project’s complexity (Table 1/Feature 3), committees established to plan within the framework of their assigned responsibilities. These committees utilized the capacity of public mobilization, NGOs, and team collaborations to maintain the dynamism of the ecosystem and ensure desirable implementation of the programmes. To enhance compatibility, the operational committees were divided into two categories: county level and neighbourhood level. The county operational committees were divided into four specialized committees to manage programmes logically and efficiently at the county level.
  Home/Non-home Support and Quarantine Committee: Responsible for mobilizing public and institutional assistance, this committee collaborated with all stakeholders, especially household health ambassadors and neighbourhood health intermediaries. Under the supervision of the Neighbourhood Operational Committee, they identified relevant households and distributed resources. The support and care teams were the operational actors of the committee, and the primary responsibility of the committee lay with NGOs.
  Tracking and Care Committee: This committee, in collaboration with the Neighbourhood Operational Committee, planned necessary measures for tracking and monitoring suspected, potential and infected individuals through electronic health record systems, neighbourhood health intermediaries, household health ambassadors and NGOs. They were responsible for active case finding, conducting COVID-19 tests on site, requesting home care teams (if necessary), disinfecting homes and training household members. The care teams (including at-home care) were the operational arm of this committee in the neighbourhoods, and the responsibility for the committee lay with the Medical Sciences Universities.
  Public Training Committee: This committee had a crucial duty to plan and coordinate public training programmes to enhance people’s knowledge and skills in dealing with COVID-19, relying on the EHHB programme and utilizing all local resources and extensive NGO participation. This was implemented through support, care and supervisory teams, and the responsibility for this committee lay with the Medical Sciences Universities.
  Monitoring and Oversight Committee: This committee was responsible for ensuring the accurate and systematic implementation of planned programmes (focussing on enforcing restrictions and health protocols) in each neighbourhood. They utilized checklists on the basis of strategies, programmes and relevant indicators to plan and carry out continuous monitoring and reporting to higher authorities.

The neighbourhood-level operational committees ensured necessary coordination among support teams, care teams and supervisory teams, providing the needed support for implementing programmes at the community level. Through the implementation of short-term-designed programmes and the establishment of collaborative and interactive networks at macro-levels (operational, training and monitoring committees) and micro-levels (support, care and supervisory teams), as well as the creation of interorganizational systems through the interaction of these networks and ICT tools (telephone system, internet portal, verbal screening), an example was created for interpreting a sequence of long-term ecosystem changes (Table 1/Feature 5).

The innovative EHHB initiative acted as an attractor (Table 1/Feature 6) for the health innovation ecosystem, and agents calibrated on the basis of it (Fig. 4: Adaptation), allowing for the ecosystem to continue its development path with a novel configuration at higher levels (Fig. 4: Emergence).

## Discussion

Although implementing the EHHB initiative outlines three critical factors for successfully managing complex projects described above, there are potential threats that could undermine future success. Threats can arise from a lack of understanding about how different components of complex development (such as EHHB) interact, the amplification of small events through feedback loops (the butterfly effect) and the emergence of unforeseen circumstances in an unplanned manner within the ecosystem. In such a case, complex developments can quickly transition from success to failure. The sustainability threats we have observed for the EHHB initiative within the Iranian health innovation ecosystem are not unique and exist for the most complex systems. The identified threats and brief explanations of their impacts in the case study of this research are presented in Table 4.

**Table 4:** Observed sustainability threats

Sustainability threats	Description
Training prioritization	In the development of complex systems, there is a great need for recognizing and understanding the necessity of training. However, in practice, sufficient attention is not usually paid to training. Iran’s health innovation ecosystem has achieved success in the EHHB project by prioritizing training and active participation of stakeholders and beneficiaries in its process. However, for future success, it is essential that education must be a continuous part of interorganizational system maintenance. That is, education is a local behaviour which has impacts on the entire project and ecosystem and improves functions and performance
Ensuring continued collaboration	In developing complex systems, the best approach is for all individuals and key stakeholders to reach an agreement and harmonize with each other. In this approach, the focus is generally on developing features or capabilities on the basis of ecosystem needs. However, when the expected functionalities of the ecosystem are delayed, and stakeholders are waiting for the promised features, they may become disappointed and hinder the necessary teamwork for development
Continuous supply of financial resources	Continuous supply of financial resources is a key factor in successfully developing of an interorganizational project. The health innovation ecosystem, through successful execution and balanced allocation of budget to various parts of the project, including operational factors, equipment and information technology costs, using primary financial resources (given the pandemic crisis and emergency conditions created), effectively controlled costs. However, after passing the tipping point, financial resources began to decline. Therefore, one of the ongoing tasks of ecosystem management is to seek financial resources to maintain operations and future development
Preventing the prevalence of temporary or unusual methods for problem-solving	When a problem arises during the development of a complex project, some actors may find temporary or alternative solutions to continue the work. Although these solutions may be helpful in the short term, if these methods are constantly and improperly accepted in interorganizational systems, they will cause further problems and widespread inconsistencies in the ecosystem. Therefore, preventing the prevalence of temporary methods and promoting permanent and standard solutions for existing issues is essential for optimizing and improving processes

Overall, the innovation ecosystem in the healthcare sector of Iran is evolving and developing. It requires regulation and coordination to connect various actors within the ecosystem to different roles and enable innovation development, particularly in paradigms such as personalized medicine, preventive and self-healing approaches, Internet of Things (IoT)-based wearable devices and the development of artificial intelligence algorithms and deep learning for disease detection. This alignment aims to enhance collaboration among these different components to advance healthcare innovation. Of course, the above issues are just examples of innovative medical paradigms, and the medical industry is constantly evolving and progressing. Furthermore, there is another importance for paradigms related to the development of innovative technologies, transformation in organizational structures and market policies.

Moreover, collaboration within an ecosystem leads to complexities arising from the interconnected network of interacting stakeholders and their diverse influences and constraints. Therefore, complexity paves the way for the successful implementation of interorganizational systems. The innovation ecosystem faces various challenges in successfully managing complex interorganizational system projects, including the need to align different processes related to institutions and their interactions with other ecosystem actors. Therefore, a small or seemingly insignificant unexpected event can disrupt the project and lead it toward failure (butterfly effect). By recognizing and embracing complexity, along with similar approaches identified in the case study of EHHB, we believe that the likelihood of success significantly increases in such projects.

### Strengths and limitations of the study

Our research stands out for its innovative use of an ecosystemic approach and using complexity and chaos theory to analyse the health innovation ecosystem’s performance and proposing transitioning from the tipping point strategies. It uniquely identifies disruption without needing to pinpoint causes, focusing on short-term changes and long-term implications. The study delves into consequences through complex adaptive systems outcomes, emphasizing the role of collaborative networks and ICT-based interorganizational systems in navigating crises and fostering healthcare innovation evolution. Our limitation in this research was the impossibility of conducting interviews with individuals who had benefitted from the EHHB project services. This was due to the lack of accurate records about these individuals. If these interviews were possible, we could add more criteria (such as culture, social capacity, and so forth) to our analyses. In the future, studying counterfactual scenarios that explore what would have happened in the absence of the EHHB programme will provide a baseline for comparison and help to establish a causal link between the programme and the observed outcome. Additionally, future research in the areas of leadership, technology and cultural adaptation can deepen our understanding of the innovation ecosystem in the healthcare sector and enhance its effectiveness in addressing the evolving needs of society in the field of health.

## Conclusions

Five principles for managing the complexity of the healthcare ecosystem are presented below, derived from the lessons learned from Iran’s healthcare innovation ecosystem and the EHHB initiative. These lessons focus on embracing the complexity of an ecosystem and lead to actions that make a difference between success and failure for an interorganizational system.

### Utilizing gap analysis to reach an agreement or a shared understanding

This principle ensures that the initial conditions for a complex project have been defined. Gap analysis has three main objectives: (1) identifying needs, (2) pinpointing current shortcomings, especially in technology and (3) aligning perceptions and viewpoints of key stakeholders within the ecosystem. Additionally, gap analysis can be used as a tool for mutual learning among agents in a complex interorganizational project. By doing so, awareness and understanding among agents about current conditions and project goals increase, and coordination and synchronization among them are facilitated. Thus, gap analysis provides a solid foundation that encompasses the progress of other aspects of the project. Without this foundation, the likelihood of project success is low. In the tipping point (the edge of chaos) that occurred in Iran’s healthcare innovation ecosystem, the key actors, having an awareness of each other’s needs and constraints, were able to implement a plan that addressed the expectations and needs of the entire ecosystem. In developing complex systems, reaching agreement and harmonization (adaptation) among all individuals is crucial and necessary. Furthermore, it appears that gap analysis provides a tool for achieving consensus in critical areas of the ecosystem, which is a vital starting point for developing a complex innovation ecosystem and controlling butterfly effects.

### Development of a collaborative and interactive management system that involves ecosystem top-level key actors

This principle emphasizes the intentional introduction of complexity in ecosystem development. In such projects, no single agent possesses the necessary comprehensive knowledge to execute the project independently. Establishing a management system that promotes interaction among team members ensures that differences and tensions are openly acknowledged, facilitating collaborative consensus building within the network. This governance structure empowers network members to address their divergences, engage in negotiations and adapt their positions as needed. Solutions often emerge through interactions among network members. Project collaboration networks are most effective when tensions arise from interactions, negotiations and collaborations among members. However, these interactions may not occur spontaneously, and ecosystem orchestrators must create conditions to foster them. Although this approach may introduce some complexity and unpredictability to the project, learning through collaborative networks leads to better solutions with fewer surprises.

### Sustaining flexibility to regulate the butterfly effects

This principle ensures that for identified risks, anticipated contingencies are in place. In complex ecosystem developments, numerous small or large events can significantly impact a programme (sensitivity to initial conditions). Complex projects can quickly spiral out of control and unexpectedly lead to undesired outcomes. In a complex interorganizational system such as the EHHB initiative, it is highly unlikely that the project will be implemented exactly as planned. Therefore, orchestrators must maintain project flexibility and ensure that collaborative teams know that issues and changes will arise. Instead of spending excessive time analysing the causes of every problem, which may sometimes be too minute to identify, project teams need to be proactive. They should anticipate future challenges, seek to comprehend the unknown aspects of each project and develop contingency plans accordingly.

### Prevent emergency solutions from becoming standard operating procedures

This principle prevents the adoption of emergency solutions that jeopardize integrating a complex interorganizational system across an ecosystem. Emergency solutions refer to configurations that actors implement to address deficiencies and inefficiencies in collaborative networks. These solutions are typically temporary measures and often indicate inefficiencies and vulnerabilities within the network. This commonly occurs when the core processes encounter issues or constraints, and instead of addressing the problem at its root, temporary and alternative measures employed. However, if these emergency solutions become standard practices, they may have a negative impact on ecosystem performance and efficiency, leading actors to rely on these temporary solutions rather than utilizing the ecosystem’s core capabilities. Therefore, this guideline emphasizes the importance for project teams to recognize and address issues and deficiencies seriously and actively work towards improving and enhancing ecosystem capabilities to eliminate the need for emergency solutions.

### Prediction of future threats to sustainability with a continuous supply of financial resources

This principle emphasizes the need for continuous budgeting in innovative and complex interorganizational project development within innovation ecosystems. Long-term budget provisions are a crucial component of interorganizational system development and foster trust among ecosystem stakeholders. Budget provisioning should be continuous throughout the entire project lifecycle. Fragmented and unstable budgets create doubt and diminish confidence in the project’s success. Therefore, financial provisions for interorganizational projects should encompass both pre-implementation and post-implementation operational phases, with a well-defined budget management plan in place.

## Data Availability

The datasets used and analysed during the current study are available from the corresponding author on reasonable request.

## Abbreviations

EHHB: Every Home is a Health Base
CAS: Complex adaptive systems
PCR: Polymerase chain reaction
NGO: Non-governmental organization

## Footnotes

1 The nationwide programme Every Home is a Health Base (EHHB) was designed and implemented in the first half of 2020, utilizing all available capacities, especially the collaboration between non-governmental organizations (NGOs) and the government. This programme aimed to provide information and control the pandemic through a bottom-up planning approach and optimal utilization of existing resources, as well as expanding collaborative and interactive networks within the health ecosystem of Iran.

2 Complex adaptive systems.
